# Morphological features in a Xhosa schizophrenia population

**DOI:** 10.1186/1471-244X-6-47

**Published:** 2006-10-27

**Authors:** Liezl Koen, Dana JH Niehaus, Greetje De Jong, Jacqueline E Muller, Esme Jordaan

**Affiliations:** 1 Ngaphakathi Group, Department of Psychiatry, University of Stellenbosch, South Africa; 2 Department of Obstetrics and Gynaecology, University of Stellenbosch, South Africa; 3 Biostatistics Unit, Medical Research Council of South Africa, Bellville, South Africa

## Abstract

**Background:**

Demonstrating an association between physical malformation and schizophrenia could be considered supportive of a neurodevelopmental origin of schizophrenia and may offer insights into a critical period for the development of this illness. The aim of our study was to investigate whether differences in the presence of minor physical anomalies could be demonstrated between schizophrenia sufferers and normal controls in a Xhosa population with a view to identifying a means of subtyping schizophrenia for use in future genetic studies.

**Methods:**

Sixty-three subjects with schizophrenia (21 sibling pairs, 1 sibship of four and a group of probands with an affected non-participating sibling (n = 17)), 81 normal controls (37 singletons and 22 sibling pairs) of Xhosa ethnicity were recruited. Each participant was then examined for minor physical anomalies using the Modified Waldrop scale. The relationship between each of the morphological features and the presence of an affected sib was examined using the Chi-squared test, followed by an intra-pair concordance analysis in the sibling pairs.

**Results:**

Gap between first and second toes was significantly more common in the affected sib pair group when compared to the non-affected sib pair group (p = 0.019) and non-affected singleton control group (p = 0.013). Concordance analysis also revealed increased concordance for this item in the affected sib pair group.

**Conclusion:**

These findings offer an intriguing possibility that in the Xhosa population, affected sib pair status may be linked to a neurodevelopmental insult during a specific period of the fetal developmental.

## Background

Schizophrenia seems to be a heterogeneous illness resulting from a complex interplay between genetic and environmental risk factors. It is currently thought that genetic factors may account for as much as 80% of the risk for developing schizophrenia and that first degree relatives of persons with schizophrenia are at higher risk of developing schizophrenia than the general population [1,2]. The risk varies according to the closeness of the relationship from approximately 8% for a non- twin sibling, to 46% for the child of two schizophrenic parents and 48% for the monozygotic twin of a schizophrenia patient [3].

Despite the apparent genetic contribution, the specific mechanism or gene has yet to be found. Indeed, models predict that schizophrenia probably has a multigenetic basis with varying contributions to the risk profile. It is thus not surprising that linkage and association studies have mapped genetic loci and tested candidate genes that appear to confer susceptibility to several chromosomal areas including 1q21-22, 1q32-34, 6p24, 8p21, 10p14, 13q32, 18p11and 22q11-13 [4,2].

In the search for the susceptibility genes it has became apparent that one possible method would be to identify a specific subgroup of schizophrenia and then attempt to locate the underlying mechanism for schizophrenia. Several approaches have been advocated for the subtyping of schizophrenia and these include demographic variables, clinical symptoms, physical characteristics and early versus late developmental insults [2]. Early developmental insults (genetic or environmental) are of particular interest in terms of the neurodevelopmental model of schizophrenia since anthropometric studies have documented multiple anomalies of the craniofacial region in schizophrenic patients [2].

An animal model of non-human primates showed that irradiation during thalamogenesis led to craniofacial abnormalities similar to those reported in schizophrenic subjects [5]. This may not be surprising since the brain and face develop from the same embryonic primordia. Furthermore, retinoic acid plays an important role during this developmental phase and retinoic acid dysregulation has been suggested as a contributing factor to the development of schizophrenia.

The aim of our study was therefore to investigate whether differences in the presence of minor physical anomalies could be demonstrated between schizophrenia sufferers and normal controls in a Xhosa population with a view to identifying a means of subtyping schizophrenia (based on the presence or absence of early developmental abnormalities) for future genetic studies. To improve the chances of a significant finding sib pairs were included, as the presence of an affected sibling not only carries an increased risk for schizophrenia but it is likely that concordant factors within a sib pair will represent shared familial or environmental factors.

## Methods

### Subjects

144 Xhosa subjects were recruited from in and outpatient services and general communities in the Western and Eastern Cape provinces of South Africa as part of an ongoing genetic study. Suitable candidates were identified by local mental health workers from all clinics within the geographic areas where Xhosa people lived. If the individuals were willing to voluntarily participate they were interviewed for suitability by the research team after written informed consent was given. Each participant had to be of Xhosa origin, suffer from schizophrenia (DSM-IV; if part of affected group) and voluntarily agree to participation. The Xhosa are an African population that form part of the Nguni language group. The relevant ethics body of the University of Stellenbosch approved this study, based on the principles of the Declaration of Helsinki.

### Assessment tools

Participants were assessed with the Diagnostic Interview for Genetic Studies (DIGS; version 2.0) [6] and the Modified Waldrop Scale [7]. The DIGS is an assessment tool structured to elicit psychotic, mood and comorbid disorders. The Modified Waldrop Scale (MWS) assesses variance in morphological features and includes the eyes, ears, oral cavity, hands and feet. Each individual was videotaped (still images available where applicable) in a standardized manner at standard camera-patient distance with a frontal and profile view of the head, palmar and dorsal views of the hands (fingers spread and unspread) and dorsal views of the feet with toes slightly spread. An experienced clinical geneticist (GDJ) evaluated each image blind to the clinical psychiatric symptom status and rated it according to the anchor points on the MWS. Only items of the MWS that were adjudged to lend themselves to reliable assessment on a still image were included. Unclear or indeterminate ratings were noted as unsure and excluded in the statistical analysis.

### Statistical analysis

The participants were divided into four groups, that of (a) group 1 – affected sib pairs (23 sib pairs [sibship of 4 provided 2 sib pairs]); (b) group 2 – 17 subjects with an affected non-participating sib; (c) group 3 – healthy individuals with no affected sib (n = 37) and (d) group 4 – unaffected sib pairs (22 sib pairs). The four groups were compared in terms of demographic variables. An overall test was done to see if the four groups were different with relation to the proportion of subjects with an abnormal feature and if significant (or marginally significant at the 5% level). Group 1 (schizophrenia sib group) was compared to each of the other three groups. Allowance was made for the correlation between the sib pairs in the analyses. A multivariate – and concordance analysis was subsequently performed.

## Results

One hundred and forty four Xhosa subjects (109 males and 35 females) were recruited. The age at interview was 36.74 years (SD 10.24; range 21–66 years) and the age of onset 22.64 years (SD 5.35; range 14–39) for the affected individuals. The mean years of schooling was 7.29 (SD 3.35) and 80% of the affected individuals were in receipt of a disability allowance.

The group was stratified into a sib pair group (n = 46 or 21 sib pairs and 1 sibship of four), a group of probands with an affected non-participating sib (n = 17), a singleton control group (n = 37) and an unaffected sib pair control group (22 sib pairs). The non-participating sib did complete a full DIGS assessment as part of an ongoing genetic study.

The sib pair group constituted 15 male-male sib pairs, 7 male-female pairs and 1 female-female pair. This included the larger sibship that was divided into two sib pairs on the basis of 1^st ^and 2^nd ^assessed (male-male pair) and 3^rd ^and 4^th ^assessed (male-female pair). The singleton group had 9 females and the group of probands with an affected non-participating sib 1 female in the group. The unaffected sib pair group consisted of 11 male-male sib pairs, 6 male-female pairs and 5 female-female pairs.

The univariate model (table 1) indicated significant differences between the affected and the non-affected sib pair groups (group 1 + 4) in terms of the presence of adherent earlobes (p = 0.0004), palatal abnormalities (p = 0.0001) and gap between first and second toes (p = 0.019). Significant differences were also detected between affected sib pairs and non-affected singleton controls (group 1 + 3) in terms of the presence of asymmetrical ears (p = 0.011) and gap between first and second toes (p = 0.013). The concordance analysis (between affected and non-affected sib pair groups) revealed significance for only one item that was also more prevalent in the schizophrenia sib pair group – gap between first and second toes (p = 0.021) (Table 2).

**Table 1:** Affected sib pairs compared to other groups: Univariate model

**Variable**	**% Abnormal**				**Chi-square**	**P**
Group*	1	2	3	4		
**N**	46	17	37	44		
**Epicantus**	10.9	23.5	24.3	31.0	5.65	0.130
**Low set ears**	35.6	29.4	30.6	17.9	2.85	0.415
**Adherent earlobes**	26.2	31.3	33.3	65.1	13.94	**0.003**
**sSibs vs grp 2**					0.37	0.543
**sSibs vs grp 3**					0.86	0.353
**sSibs vs grp 4**					12.59	**0.0004**
**Malformed ears**	9.1	0.0	8.1	2.3	-	-
**Palatal abnormalities**	48.6	41.7	36.0	96.3	19.13	**0.0003**
**sSibs vs grp 2**					0.10	0.750
**sSibs vs grp 3**					0.69	0.407
**sSibs vs grp 4**					15.29	**0.0001**
**Tongue abnormalities**	10.3	11.1	17.9	19.5	1.71	0.635
**5**^**th**^**finger variations**	17.8	25.0	21.6	18.6	0.35	0.950
**Transverse palmar crease**	2.2	0.0	2.8	7.0	-	-
**Third toe abnormalities**	0.0	0.0	0.0	0.0	-	-
**Syndactalia**	19.6	6.3	25.0	18.6	3.86	0.277
**Asymmetrical ears**	9.1	17.7	32.4	2.3	12.68	**0.005**
**sSibs vs grp 2**					0.64	0.422
**sSibs vs grp 3**					6.51	**0.011**
**sSibs vs grp 4**					2.25	0.133
**Gap between toes**	31.1	20.0	5.6	7.0	7.43	0.059
**sSibs vs grp 2**					0.55	0.460
**sSibs vs grp 3**					6.16	**0.013**
**sSibs vs grp 4**					5.52	**0.019**

*Group 1 sib pairs with schizophrenia (sSibs)

Group 2 probands with an affected non-participating sib

Group 3 singleton controls, unaffected

Group 4 sib pairs, unaffected

**Table 2:** Modified Waldrop items: concordance findings for affected and non-affected sib pairs

								**STATISTICS**	
**ITEM**	**GROUP**	**CONCORDANT FOR ABSENCE OF DYSMORPHOLOGY**		**DISCONCORDANT**		**CONCORDANT FOR PRESENCE OF DYSMORPHOLOGY**		**CHI SQUARE**	**p**

**Low set ears**	sSibs	11	50.0	7	31.8	4	18.2	2.09	0.35
	unafSibs	13	72.2	3	16.7	2	11.1		
**Epicanthus**	sSibs	19	82.6	3	13.0	1	4.4	3.94	0.14
	unafSibs	11	55.0	7	35.0	2	4.7		
**5**^**th **^**finger**	sSibs	17	77.3	2	9.1	3	13.6	7.37	**0.025**
	unafSibs	14	66.7	7	33.3	0	0.0		
**Adherent ear lobe**	sSibs	11	57.9	5	26.3	3	15.8	11.42	**0.003**
	unafSibs	2	9.5	11	52.4	8	38.1		
**Malformed ears**	sSibs	17	80.9	4	19.1	0	0.0	2.17	0.14
	unafSibs	20	95.2	1	4.8	0	0.0		
**Asymmetrical ears**	sSibs	17	80.9	4	19.1	0	0.0	2.17	0.14
	unafSibs	20	95.2	1	4.8	0	0.0		
**abnormalities**	sSibs	6	85.7	1	14.3	0	0.0	1.45	0.25
	unafSibs	12	63.2	7	36.8	0	0.0		
**Gap between 1**^**st **^**and 2**^**nd **^**toe**	sSibs	12	54.6	6	27.3	4	18.2	7.75	**0.021**
	unafSibs	18	85.7	3	14.3	0	0.0		
**Palatal abnormalities**	sSibs	4	25.0	8	50.0	4	25.0	16.62	**0.0002**
	unafSibs	0	0.0	0	0.0	9	100		
**Syndactalia**	sSibs	15	65.2	7	30.4	1	4.4	0.021	0.99
	unafSibs	14	66.7	6	28.6	1	4.8		
**Transverse palmar crease**	sSibs	19	95.0	1	5.0	0	0.0	2.17	0.14
	unafSibs	20	91.4	2	9.6	0	0.0		

sSibs = group 1 = sib pairs with schizophrenia

unafSibs = group 4 = sib pairs, unaffected

The multivariate analysis revealed that gender was a significant variable in the model for asymmetrical ears (males, p = 0.008), syndactaly (females, p = 0.0004) and gap between first and second toes (males, p = 0.009), but including gender in the model did not change the results for the morphological features, i.e. gender did not confound the relationship of the groups with the morphological features.

## Discussion and conclusion

This is the first study to report on the morphological features as measured by the Modified Waldrop scale, in an African Xhosa schizophrenia sib pair population. Minor physical anomalies were noted in all of the items measured by the Modified Waldrop Scale. The most striking finding is that of significantly more subjects with a gap between the toes in the affected sib pair group and the affected sib with non-participating sib group. Although other significant differences were detected, only gap between toes shows a consistent pattern as would be expected if a morphological abnormality contributed to the affected status. Given the increased morbid risk for a sib of a schizophrenic this finding may suggest that this anomaly represents a developmental period of specific importance in familial cases of schizophrenia. This finding differs from that of Gourion et al. [8] who reported a higher rate of low set ears (OR = 11.9), cleft palate (OR = 8.5), curved 5th finger (OR = 3.6) and syndactaly (OR = 3.6) in schizophrenia patients compared to controls. However, it still supports the possible role of brain developmental processes in the risk for schizophrenia and the differences may be linked to population specific risk factors for schizophrenia.

The interpretation of the data is subject to the following methodological problems: the sample size is small and type 2 errors could have occurred. It could be that the unaffected siblings from the singleton group have not yet passed through the vulnerability phase for schizophrenia and this could have an influence on the group distributions. It would be of value to also incorporate a control group in as far as some of the items that were either concordant or significantly different between the groups might represent a less familial risk period for the development of schizophrenia.

Nevertheless, the development of the distal extremities takes place between day 50 and 70 of gestation, in the same time frame as the development of the thalamus. Indeed, Andreasen [9] has proposed that schizophrenia is a neurodevelopmental illness which arises because of a defect in prefrontal-thalamic-cerebellar circuitry. The thalamus is a key relay nucleus that modulates both motor and cognitive coordination. Deficits have been described in both these modalities [10-12].

There has also been some structural evidence for thalamic abnormality in schizophrenia. Several neuropathological studies, including the study of Pakkenburg [13] have shown decreased neuronal density and reduced volume of the medial dorsal nucleus of the thalamus in the absence of gliosis, again suggesting a neurodevelopmental rather than neurodegenerative process. Four magnetic resonance studies [9] showed reduced thalamic size and two PET studies [14,15] showed abnormal thalamic activity in patients suffering from schizophrenia. Thus, apart from the possible link between our morphological findings and thalamic development, there also seems to be a clear link between the thalamus and the pathophysiology of schizophrenia.

These findings offer an intriguing possibility that in the Xhosa population, affected sib pair status may be linked to a neurodevelopmental insult during a specific period of the fetal developmental. Follow-up studies in an independent sample will investigate these and other morphological abnormalities that could narrow the risk period and may offer some insights into the susceptibility of schizophrenia. Furthermore, in the light of the previously reported finding that the core symptoms of schizophrenia in the Xhosa population is similar to that found in other ethnic populations [16] it would be very interesting to compare our specific minor physical anomalies presence findings with those previously reported in Caucasian populations.

## Competing interests

The author(s) declare that they have no competing interests.

## Authors' contributions

LK and DJHN conceived of and designed the study and drafted the manuscript. JE clinically evaluated the participants. GDJ did the morphological evaluation of the participants. EJ performed the statistical analysis. All authors read and approved the final manuscript.

## Pre-publication history

The pre-publication history for this paper can be accessed here:


